# Extension of the right internal thoracic artery with the radial artery in extensive re-do coronary artery bypass grafting

**DOI:** 10.1186/1749-8090-8-173

**Published:** 2013-07-12

**Authors:** Felix Fleissner, Fabio Ius, Axel Haverich, Issam Ismail

**Affiliations:** 1 Division of Cardiac, Thoracic, Transplantation and Vascular Surgery, Hannover Medical School, Hannover, Germany

## Abstract

**Background:**

Patients with extensive coronary artery disease often require re-do coronary artery bypass grafting. However, autologous bypass material is sometimes sparse. Since long term patency of arterial graft material is superior to venous bypass grafting, we developed a technique to perform re-do total arterial coronary artery bypass grafting extending the right internal thoracic artery (RITA) with the radial artery (RA) in an end to end fashion to gain the needed length in patients with and without an open left thoracic artery (LITA).

**Methods:**

We performed this approach in 27 consecutive patients (age: 67.93 ± 7.51 years). Data was analyzed retrospectively. 19 operations were first re-op, 6 were second re-op and two were third re-op procedures.

**Results:**

Cardiopulmonary bypass time was 115.42 minutes (±31.92 minutes) with one OPCAB procedure, and clamp time was 55.09 minutes (±22.41 minutes) excluding 10 procedures performed on beating heart. Bypass grafting included the RCA, Cx and LAD. An average of 1.96 anastomoses were performed in each patient. Complication rate was low with one intra-operative apoplexy and one prolonged wound healing after harvest of the radial artery. One patient needed long term pulmonary assist. There was no intra-operative or early postoperative death.

**Conclusion:**

The operational technique of elongation of the internal thoracic artery with the radial artery proved to be safe and feasible with acceptable operation times for a re-do procedure. We recommend this as an additional option to existing methods to perform a complete arterial revascularization mainly in patients with open left internal thoracic artery to LAD bypass.

## Background

Coronary artery bypass grafting remains the gold standard for patients with multivessel disease and left main coronary artery disease [1]. The efficacy of coronary artery bypass grafting for patients with ischemic heart disease is dependent on the selected conduit. However, autologous bypass material is sparse, especially in re-do coronary artery bypass grafting cases. Meta-analyses suggest superiority of the radial artery over the saphenous vein concerning patency [2,3]. RA patency rates range from 83% to 93% at one to seven years after operation [4]. We therefore developed a technique to perform re-do total arterial coronary artery bypass grafting extending the right internal thoracic artery (RITA) with RA in an end to end fashion to gain the needed length.

## Methods

### Patients

27 patients (25 male, 2 female, mean age 67.93 years) operated between 2005 and 2011 were enrolled in this observational study. All of them had received an extension of the right mammalian artery using the radial artery in our clinic. All of them were re-do cases (19 operations were first re-op, 6 were second re-op and two were third re-op procedures). The logistic STS Score for mortality was 9.08 (±5.14, range 3.39-24.7) (Table 1). Two patients received previous venous bypass grafts only. We chose this approach in only a minority of patients at the surgeon’s preference. In total, we performed 6025 CABG cases during this time period with approximately 5 percent cardiac re-do cases. In the other cases, we used either venous bypass grafts and or, if feasible, LITA or RITA grafts.

**Table 1:** Patient’s characteristics

***Variable***		
Total patients n (100%)	24	(100)
Age yrs (SD)	67.93	(7.51)
Sex male n (%)	22	(81.48)
BMI (SD)	27.21	(3.46)
LV function % (SD)	50.27	(12.9)
Previous PTCA n (%)	9	(33.33)
Previous MI n (%)	9	(33.33)
Previous CABG n (%)	24	(100)
First re-op n (%)	19	(70.37)
Second re-op n (%)	6	(22)
Third/more than third re-op n (%)	2	(7.41)
Elective n (%)	26	(96.30)
Emergency n (%)	1	(3.70)
STS Score SD	9.08	(5.14)

### Surgical technique

**Figure 1:**
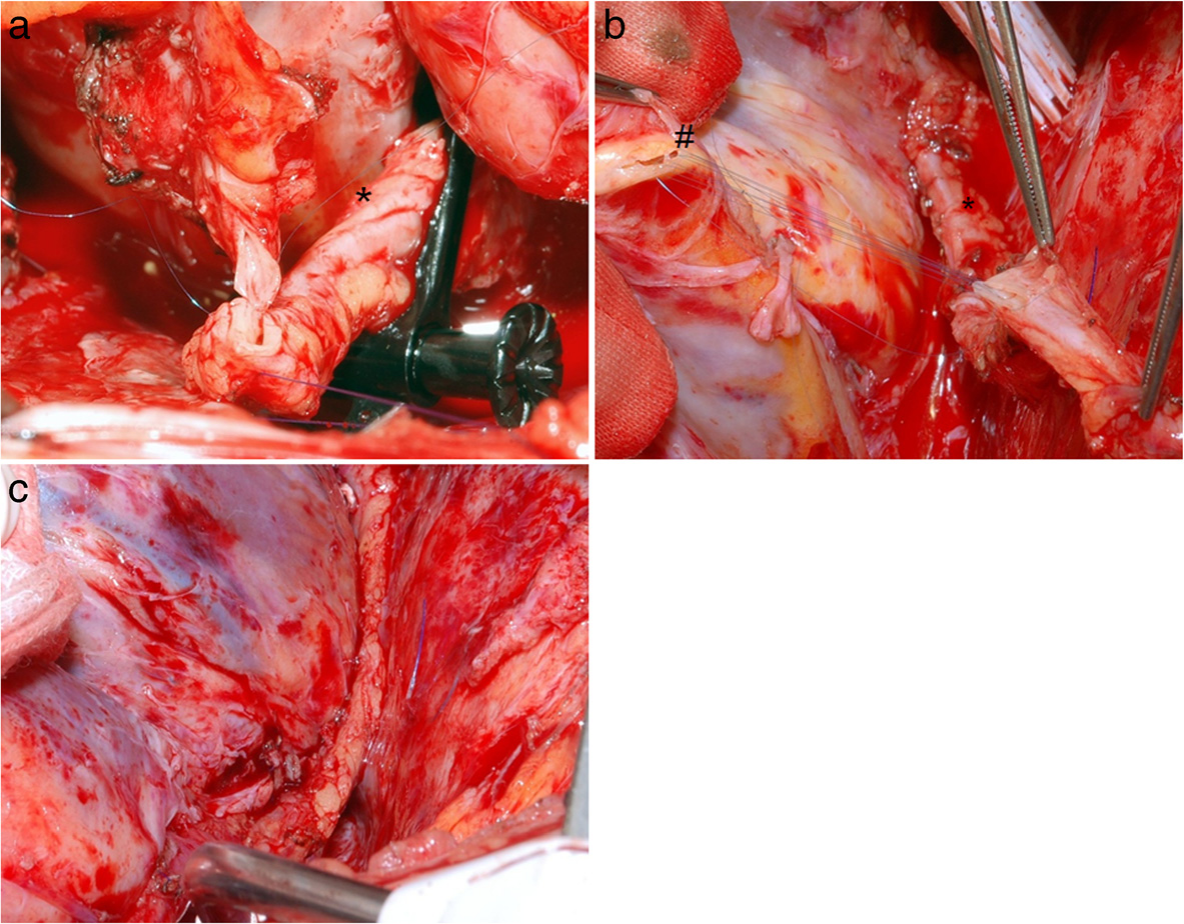
**In-situ view of the extended RITA-RA bypass graft. a**, (*) Right thoracic artery anastomosis (RITA) to (+) radial artery (RA) end to end anastomosis. **b**, extended (*) RITA-RA to (#) RIVP anastomosis. **c**, The RITA-RA graft in situ. Of note the RITA-RA anastomosis is placed intra-pericardial for better protection.

All patients underwent ultrasound evaluation and a modified Allen test prior to operation of the RA. RA was harvested in a routine fashion using a no-touch technique in combination with low energy cautery. Anti-spasm prophylaxis consisted of 1 mg/ml papaverine applied topically. The surgical approach was a median sternotomy using a oscillating saw as standardized for re-do cases in our clinic, except for one patient who received a limited clamp shell approach. The RITA was prepared, left in situ and extended using the radial artery in an end to end fashion with a 7/0 suture (see Figure 1). We tried to perform the anasotmosis within the pericardium for further protection. Special care was undertaken to stay clear of the sternum for easier future access (5). The length of the extended RITA was sufficient to reach all three major vessels, and to perform up to three sequential bypasses if necessary. We generally used cardiopulmonary bypass except for one patient who was operated on during fibrillating heart. The aorta was clamped in 16 of the 27 patients. Two patients received a concomitant aortic valve replacement, both due to an aortic stenosis.

### Follow up

End point was the occurrence of major cardiac event, defined as follows: 1) cardiogenic death; 2) myocardial infarction reported by patient or hospital admission for myocardial infarction reported by cardiologist; and 3) the need for revascularization (repeat operation or angioplasty reported by patient or cardiologist through follow up period.

### Statistical analysis

Continuous data are expressed by mean + − SD. Categorical numbers are summarized by reporting the percentages. Cumulated survival was estimated using the Kaplan and Meier method. Statistical analysis was performed using SPSS 20 package (SPSS Inc, Chicago, Il, USA).

### Ethics

This retrospective study was approved by the Ethics Committee of Hanover Medical School and all patients gave their consent.

## Results

**Figure 2:**
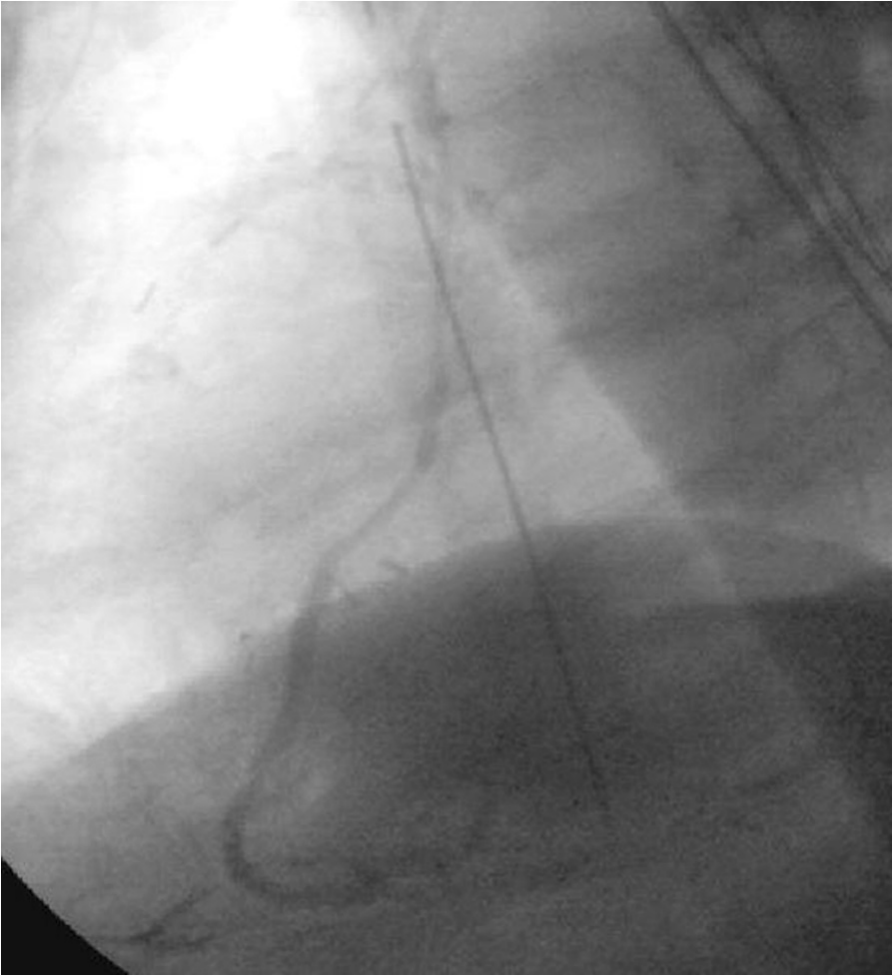
Postoperative coronary angiogram of the RIMA-Rad-RIVP-PLA2 bypass graft performed in patient number 17.

Total operation time was 275.25 minutes (±59.62 minutes), cardiopulmonary bypass time was 115.42 minutes (±31.92 minutes) with one OPCAB procedure, and clamp time was 55.09 minutes (± 22.41 minutes), respectively. One patient received an IABP pre-operative and one patient postoperative. 9 procedures were performed on beating heart and one as an OPCAB procedure. An average of 1.96 anastomoses were performed in each patient (see Table 2). A detailed list of the performed anastomoses is found in Table 3. We were able to gain sufficient length with the extended RITA to perform up to four sequential bypasses to branches of the right coronary artery, the circumflex artery and the LAD, respectively. There were no intra-or early postoperative deaths. In average, the patients remained on the ICU for 3.14 days (±5.9 days), and left the primary hospital after an average of 9.4 days (±4.4 days). One patient suffered from an intra-operative stroke. One patient displayed ST-elevations the first postoperative day. Emergent heart catheterization showed only a minor stenosis of the RITA-RA anastomosis and no de-novo stenoses of the native coronaries (Figure 2). Postoperative course was uneventful, except for occurrence of atrial fibrillation in two patients and prolonged ventilation in one patient. One patient had a donor site infection which was treated by intravenous antibiotics. No patient developed ischemic forearm, hand or finger complications.

**Table 2:** Operational details and early postoperative complications

***Operational details***		
Numbers of anastomosed vessels n (SD)	1.93	(0.71)
Opcab procedures n (%)	1	(3.7)
Beating heart n (%)	9	(33.33)
Cardiopulmonary bypass min (SD)	115.42	(31.92)
Clamp time min (SD)	55.09	(22.41)
*complications*		
Stroke n (%)	1	(3.7)
Radial artery harvest site infection n (%)	1	(3.7)

**Table 3:** Overview of the performed bypass grafts in all patients

***Performed bypass grafts***	**n**	**Numbers of anastomoses**
RITA-Rad-LAD	4	1
RITA-Rad-PLA	1	1
RITA-Rad-RIVP	2	1
RITA-Rad-RCA	1	1
RITA-Rad-RIVP-PLA	9	2
RITA-Rad-PLA-RCA	3	2
RITA-Rad- RIVP-LAD	2	2
RITA-Rad-RIVP-RIM	1	2
RITA-Rad-PLA-PLA-RIVP	2	2
RIMA-Rad-RIVP-PLA1-LAD	1	3
RITA-Rad-RCA-PLA-RIM	1	3

Follow up was complete. Median follow up was 41 months (ranging from 12 to 85 months). There was no death during follow up. Only 2 patients had a coronary angiogram during follow up due to angina or non- ST elevation myocardial infarction. In both patients, the extended RITA graft was patent, however in one case with a minor stenosis. There was a significant reduction in reported Angina. Pre-operative, 44.4% (n = 12) of all patients reported angina class CCS 1–2, and 44.4% (n = 12) of patients were CCS class 3–4 (3 were unknown). Postoperative, only 7.4% (n = 2) of patients were classified as CCS 3–4 and 77.8% (n = 21) were classified CCS 1–2 (4 patients were unable to classify their status of angina). The two patients remained in angina class II-IV despite an extensive antianginal medication. However, due to non cardiac related severe illnesses, no further intervention was undertaken in these patients.

## Discussion

By our case series we could show that extending the RITA using the radial artery is sufficient in length to re-vascularise all three major vessel areas. Early to midterm follow up revealed a low rate of necessary coronary angiograms and PTCAs due to angina and no death during follow up. Angina pectoris was significantly reduced in our patients. There was no intra-operative and no follow up mortality, even though the average STS score was high. Our mortality rate of 0% and complication rates are lower then those reported in the literature for re-do CABG operations [4], however this case series only includes a very limited number of carefully selected patients. In older series the observed mortality has been reported to be much higher ranging from 6.9 to 9.2% [5,6].

Limitations to our study are the retrospective approach and lack of a post-operative angiogram or computed angiography in all patients. However, there is so far no routine post- CABG angiogram established at our centre. Concerns about long term patency of the rather long bypass grafts have to be addressed in the future. Avoiding the use of vein grafts improves long term durability and limits donor site complications [7]. And, for especially the third-re-do cases autologous vein graft material can be simply inexistent. Re-do cardiac surgery using the radial artery is well established with good results [8]. However, usually the RA is anastomosed to the proximal aorta showing acceptable results with a low mortality and postoperative complications, including a very low rate of spasm of the RA [8]. We do believe that using the extension of the radial artery avoids the proximal aortic anastomosis, which especially in re-do cases and calcified aortas can be a problem [9]. An interesting technique by Pehkonen et al. already introduced the anastomosis of the RA to the unharvested RITA [10]. However, the gained length of the RA alone anastomosed to the unharvested RITA is not sufficient to revascularize distal coronaries. Also, collateral flow through the RITA remains a problem. A possible weak point of this composite and sequential graft is that flow is dependent on a sequential in situ RITA graft. However, due to the expected longer patency over venous graft and our experience of a low complication rate early and midterm postoperative, we believe that this concept is safe and feasible for selected patients.

The length of the RITA-RA graft is also usually long enough to avoid crossing the midline beneath the sternum to avoid damage during a possible re-re-do operation.

## Conclusion

In conclusion, we present a case series of re-do CABG patients with open LIMA-LAD bypass with excellent results using an extension of the RITA with the RA.

## Abbreviations

(CABG): Coronary artery bypass grafting; (RA): Radial artery; (RITA): Right internal thoracic artery; (LITA): Left thoracic artery.

## Competing interests

The authors declare that they have no competing interests.

## Authors’ contributions

FF and II designed the study, performed the statistical analysis and wrote the manuscript, AH and FI revised the manuscript and gave valuable contributions to the study. All authors read and approved the final manuscript.
